# Hemothorax complicating rheumatoid arthritis

**DOI:** 10.4103/1817-1737.78433

**Published:** 2011

**Authors:** Mahesh Prakash, Subramaniyan Ramanathan, Surjit Singh, Niranjan Khandelwal

**Affiliations:** *Department of Radiodiagnosis, Post Graduate Institute of Medical Education and Research, Chandigarh, India*; 1 *Department of Internal Medicine, Post Graduate Institute of Medical Education and Research, Chandigarh, India*

A 50-year-old man presented with shortness of breath and fever for a duration of 7 days. He was a known case of rheumatoid arthritis on treatment for the past 7 years from rheumatology clinic of our institute. On clinical examination he was drowsy and hemodynamically stable. Respiratory rate was 25/min with bilateral decreased breath sounds and fine crepitations. Chest radiograph showed right-sided pleural effusion. Laboratory investigations were performed [Table 1]. Ultrasound-guided diagnostic thoracentesis showed bloody aspirate. Pleural fluid analysis revealed hematocrit, 25%; white blood cell count, 4000; protein, 3.3 g/dL; LDH, 400 U/L; and glucose, 80 mg/dL. Contrast enhanced computed tomography of chest was carried out [Figures 1 and 2].

**Table 1:** Laboratory study results

Laboratory parameter	Observed value
Hemoglobin	9 g/dL
Hematocrit	35%
Total white blood cells	16,000/mm^3^
Platelet	300,000/mm^3^
Erythrocyte sedimentation rate	68 mm at 1 h
Random blood glucose	120 mg/dL
Lactate dehydrogenase	480 U/L
Prothrombin time	12 s
Activated partial thromboplastin time	33 s
Anti nuclear antibody	Positive (diffuse type)
C reactive protein	48 mg/dL
Rheumatoid factor latex agglutinin test	Positive

**Figure 1:**
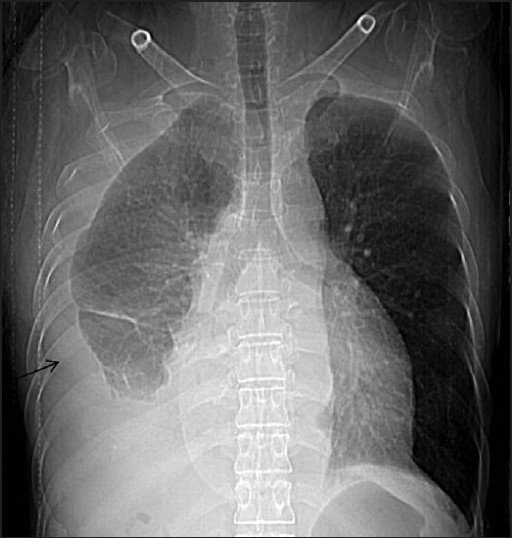
Computed tomography scanogram showing right pleural effusion

**Figure 2:**
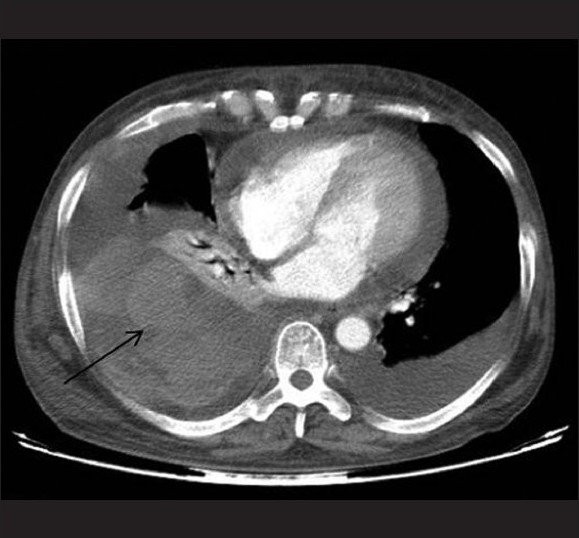
Computed tomography scan of chest shows right-sided hemothorax, left pleural effusion, and pericardial effusion

## Questions

1. What are the findings on the computed tomography of the chest?
2. What is the differential diagnosis?

## Answers

1. Large right pleural effusion with hyperdense contents suggestive of hemothorax with mild left pleural and pericardial effusion was seen.
2. Common causes of hemothorax include trauma, infections, coagulopathy, malignancy, and vascular abnormalities.

## Course of the Patient

The diagnosis of spontaneous hemithorax was made. Chest tube was placed which drained approximately 1 L of blood immediately and two units of whole blood transfused with close monitoring of vitals. In the next 4 h 500 ml of blood drained. The patient was conservatively managed as his hemodynamic status was stable. Pleural fluid analysis was negative for malignant cells. Abdominal fat pad biopsy turned out to be positive for amyloid (Congo red positive).

## Discussion

Rheumatoid arthritis is a common connective tissue disorder of autoimmune etiology affecting multiple organ systems presenting with symmetric polyarthralgia. Pleuropulmonary manifestations of RA are commonly seen in males and include pleural effusion, pneumonitis, pleuropulmonary nodules, interstitial fibrosis, arteritis, bronchiectasis, and amyloidosis. Pleural effusion is the most common chest manifestation of RA and is usually small, bilateral, and asymptomatic. It is an exudative effusion with raised LDH, increased protein, and decreased glucose.[1]

Spontaneous hemothorax is an unusual complication of RA the exact incidence of which is not known and its pathogenesis is not well understood. Hemothorax is defined as pleural fluid hematocrit >50% of blood hematocrit. Common causes of hemothorax include trauma, infections, coagulopathy, malignancy, and vascular abnormalities.[2] In our patient as coagulopathy and malignancy were ruled out, other rare cause of hemothorax, amyloidosis (secondary) was considered. A primary mechanism of bleeding in amyloidosis is coagulation abnormalities either isolated or multiple factor deficiencies.[3] But literature search revealed a few case reports of bleeding in amyloidosis secondary to amyloid infiltrate of vascular and perivascular connective tissues in the absence of coagulopathy.[4,5] This amyloid infiltration leads to increased fragility of blood vessels leading to poor hemostasis.[4] In our case, there was significant hemothorax in the patient with long standing RA with normal coagulation tests and no other predisposing factors. Abdominal fat pad biopsy was strongly positive for amyloid and was assumed to be the cause of hemothorax. Basoglu[6] reported one case of spontaneous hemo-pneumothorax in a young patient with rheumatoid lung disease. The cause of hemopneumothorax in this patient was bleeding from the lingular branch of pulmonary artery with adjacent parenchymal necrosis.

Treatment options reported include splenectomy, aggressive treatment of the primary disease, melphalan, prednisone, tumor necrosis factor (TNF-α), and interlukins (IL-1).[3] Spontaneous remissions have also been reported. In our case hemothorax improved after chest tube drainage and the patient was clinically stable. He is now managed with disease-modifying anti-rheumatic drugs (DMARDs) and is on close follow-up.

In conclusion, hemothorax is very uncommon in a case of RA without any predisposing factors or coagulation abnormalities. One should consider the possibility of secondary amyloidosis in such long standing cases of RA.
